# Proteomics analysis on lipid metabolism in *Elaeis guineensis* and *Elaeis oleifera*

**DOI:** 10.1016/j.dib.2020.105714

**Published:** 2020-05-16

**Authors:** Benjamin Yii Chung Lau, Mohd Din Amiruddin, Abrizah Othman

**Affiliations:** Malaysian Palm Oil Board

**Keywords:** Proteomics, Oil palm, *Elaeis guineensis*, *Elaeis oleifera*, Fatty acid biosynthesis, Lipid metabolism

## Abstract

Proteome data was obtained from the fruit mesocarps of the two oil palm species, namely, the African *Elaeis guineensis* (commercial tenera or commonly known as D x P and MPOB-Nigerian tenera) and the South American *Elaeis oleifera*. Total proteins were extracted from randomly selected fruitlets and subjected to proteomics characterisation by means of liquid chromatography mass spectrometry. Number of proteins identified, the grouping of the biological replicates from five developmental weeks after anthesis, and the localisation of gene corresponded to the detected proteins on the oil palm chromosomes, were presented. A total of 4,116, 4,210 and 4,081 proteins were found in commercial tenera and MPOB Nigerian tenera for *Elaeis guineensis*; and *Elaeis oleifera*, respectively. Principal component analysis showed two distinct clusters that corresponded to *Elaeis guineensis* and *Elaeis oleifera.* Collectively, genes that corresponded to the identified proteins were found to be located in all 16 oil palm chromosomes. A total of 59 proteins from *Elaeis guineensis* and *Elaeis oleifera* were down-regulated for >5-fold change during the peak of lipid biosynthesis compared to the onset. The same comparative analysis revealed that 66 proteins were up-regulated for >5-fold change. About 60.0% of the observed proteins were involved in catalytic activity while 28.5% were associated with redox reaction. Based on same datasets, the tricarboxylic acid cycle and 5-hydroxytryptamine degradation pathways were found to be enriched the most (>36-fold change). These data can be used to support the oil palm gene model validation and lipid metabolism research, particularly in the areas of oil yield and quality. The tabulated protein lists of identified proteins and their expression changes from these varieties were provided as supplementary files. Raw MSF and mzid files for all the oil palm species were deposited in the ProteomeXchange (PXD017436).

Specifications table

**Table:** 

Subject	Agriculture Science
Specific subject area	Plant lipid metabolism
Type of data	Figure Proteome data
How data were acquired	Instruments: EASY-nano liquid chromatography (EASY-nLC) 1200 System, Q Exactive Plus Hybrid Quadrupole-Orbitrap (Thermo Scientific) Software: Proteome Discoverer 2.2 (Thermo Scientific)
Data format	Raw and analysed
Parameters for data collection	Oil palm mesocarps of *Elaeis guineensis* (commercial tenera or D x P), MPOB-Nigerian tenera and *Elaeis oleifera* of five developmental weeks after anthesis were used for protein extraction.
Description of data collection	Total protein was extracted from oil palm fruit mesocarps. The total protein was further digested with trypsin to generate peptides. EASY nLC 1200 System was used for peptide separation while mass spectra were acquired using Q Exactive Plus System. Raw data was analysed using Proteome Discoverer 2.2.
Data source location	Hulu Paka (Terengganu) and Kluang (Johor), Malaysia
Data accessibility	Raw MSF and mzid files were deposited in ProteomeXchange (PXD017436). Processed datasets are with the article
Related research article	Benjamin Yii Chung, Lau, James D Morton, Santanu Deb-Choudhury, Stefan Clerens, Jolon M Dyer and Umi Salamah Ramli. Differential expression analysis of oil palm fatty acid biosynthetic enzymes with gel-free quantitative proteomics. Journal of Oil Palm Research, Volume 29, 23-34, 2017.

## Value of the data

• Proteome comparison of three oil palm populations from the two species (*Elaeis guineensis* and *Elaeis oleifera*) are presented for the first time.
• The data can be used to understand the regulation mechanism of oil yield and high-value fatty acids.
• The data can be used to identify differentially expressed proteins and proteins that characterised these populations and the two species.
• The data can be integrated to quantitative genetic analysis and support gene model validation.

## 1 Data

**Fig. 1:**
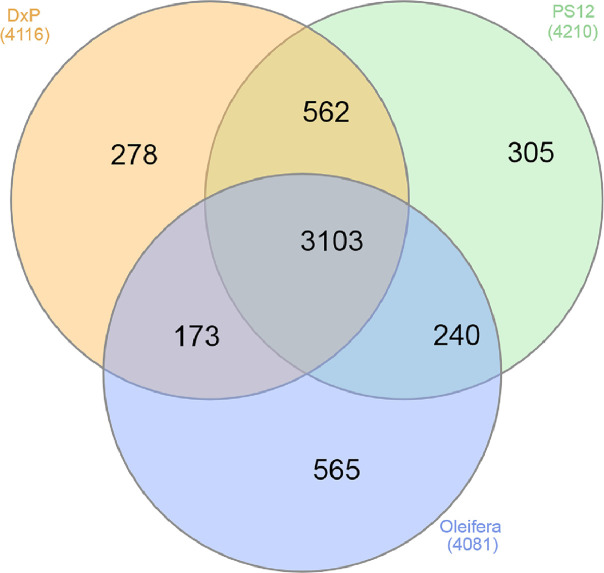
Venn diagram of proteins identified from oil palm mesocarps of *Elaeis guineensis* (commercial tenera), MPOB-Nigerian tenera and *Elaeis oleifera*. The data was obtained using SEQUEST-HT protein search engine.

**Fig. 2:**
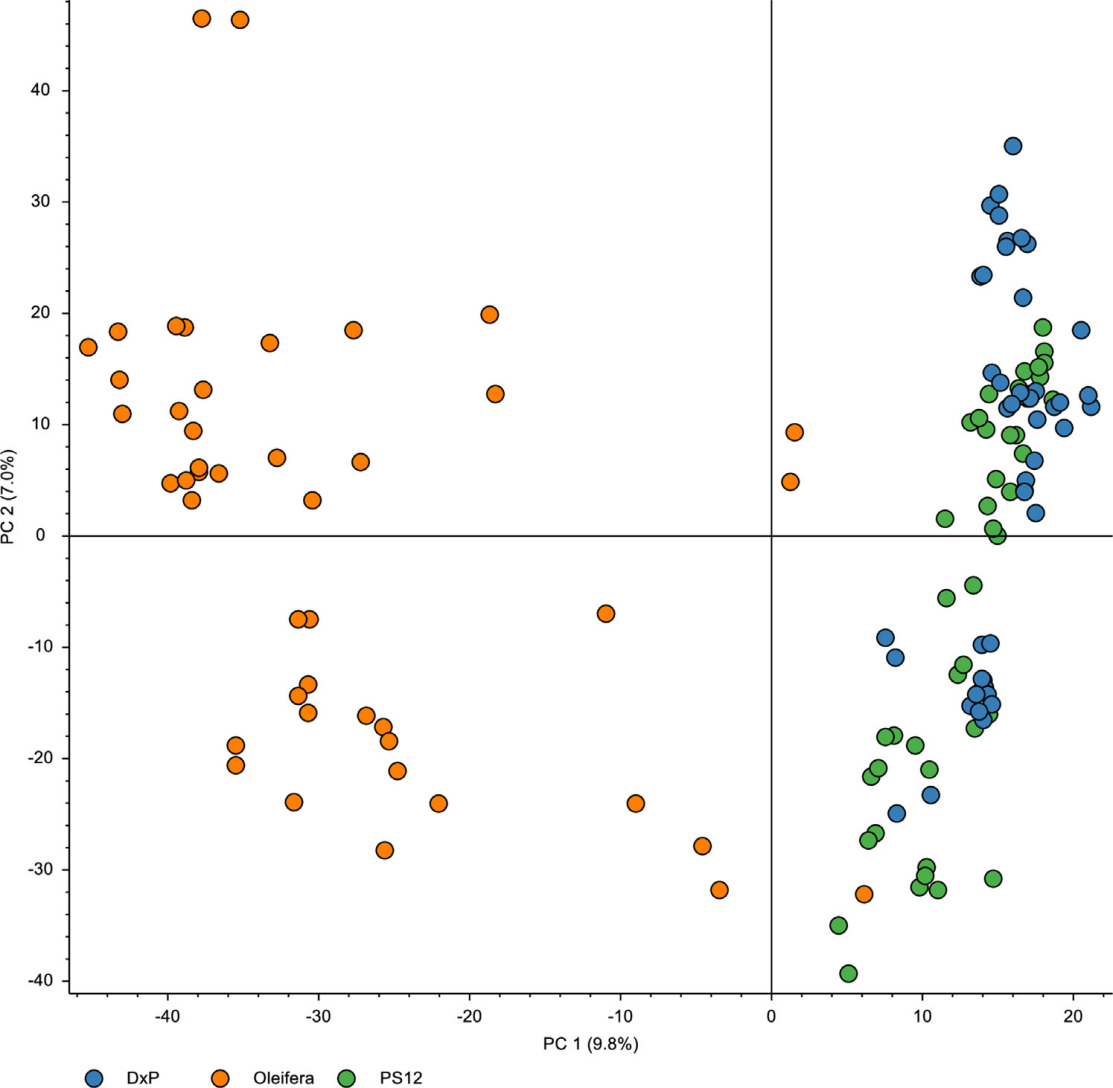
Principal component analysis plot representing proteomics data from the comparative analysis of commercial *Elaeis guineensis* (commercial tenera), MPOB-Nigerian tenera and *Elaeis oleifera*. The plot displays two distinct clusters.

**Fig. 3:**
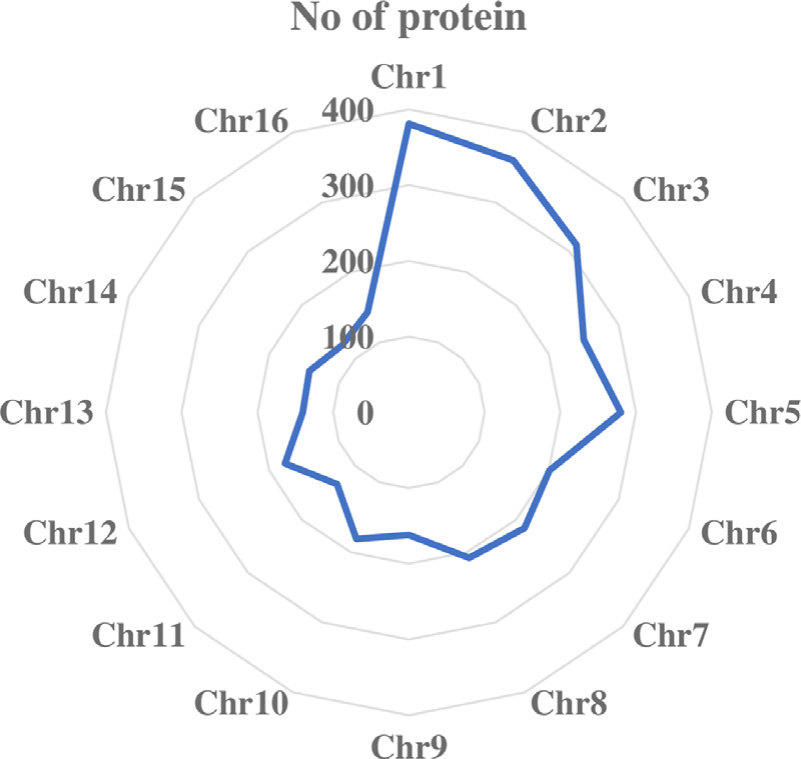
Chromosome location of the identified proteins from commercial *Elaeis guineensis* (commercial tenera), MPOB-Nigerian tenera and *Elaeis oleifera*.

Proteome data for each of the oil palm populations was generated using shotgun proteomics approach. In total, 4,116, 4,210 and 4,081 proteins were identified in *Elaeis guineensis* (commercial tenera or D x P), MPOB-Nigerian tenera and *Elaeis oleifera*, respectively (S1). A total of 3,103 proteins were found to be present in all populations (Fig. 1). 278 proteins were unique to commercial tenera while MPOB-Nigerian tenera had 305 unique proteins. 565 proteins were only identified to *Elaeis oleifera.* Grouping of the biological replicates using principal component analysis was presented in Fig. 2. Gene location on the 16 oil palm chromosomes [1] that corresponded to the identified proteins was shown in Fig. 3. The raw data had been deposited in the ProteomeXchange (PXD017436) for reference.

The data obtained from the label-free quantitative proteomics analysis was then applied to calculate the fold change at a normalized ratio for peak of lipid biosynthesis (16^th^ and 18^th^ week after anthesis) to onset of lipid biosynthesis (12^th^ week after anthesis). Only observed proteins involved in lipid metabolism process with changes in abundances from *E. guineensis* and *E. oleifera* were determined (S2). The statistic analysis revealed 28 (16th week after anthesis) and 31 significant proteins (18th week after anthesis) were down-regulated for >5-fold change when compared to the onset of lipid biosynthesis. A total of 29 and 37 proteins were up-regulated for >5-fold change relative to the onset of lipid biosynthesis.

**Fig. 4:**
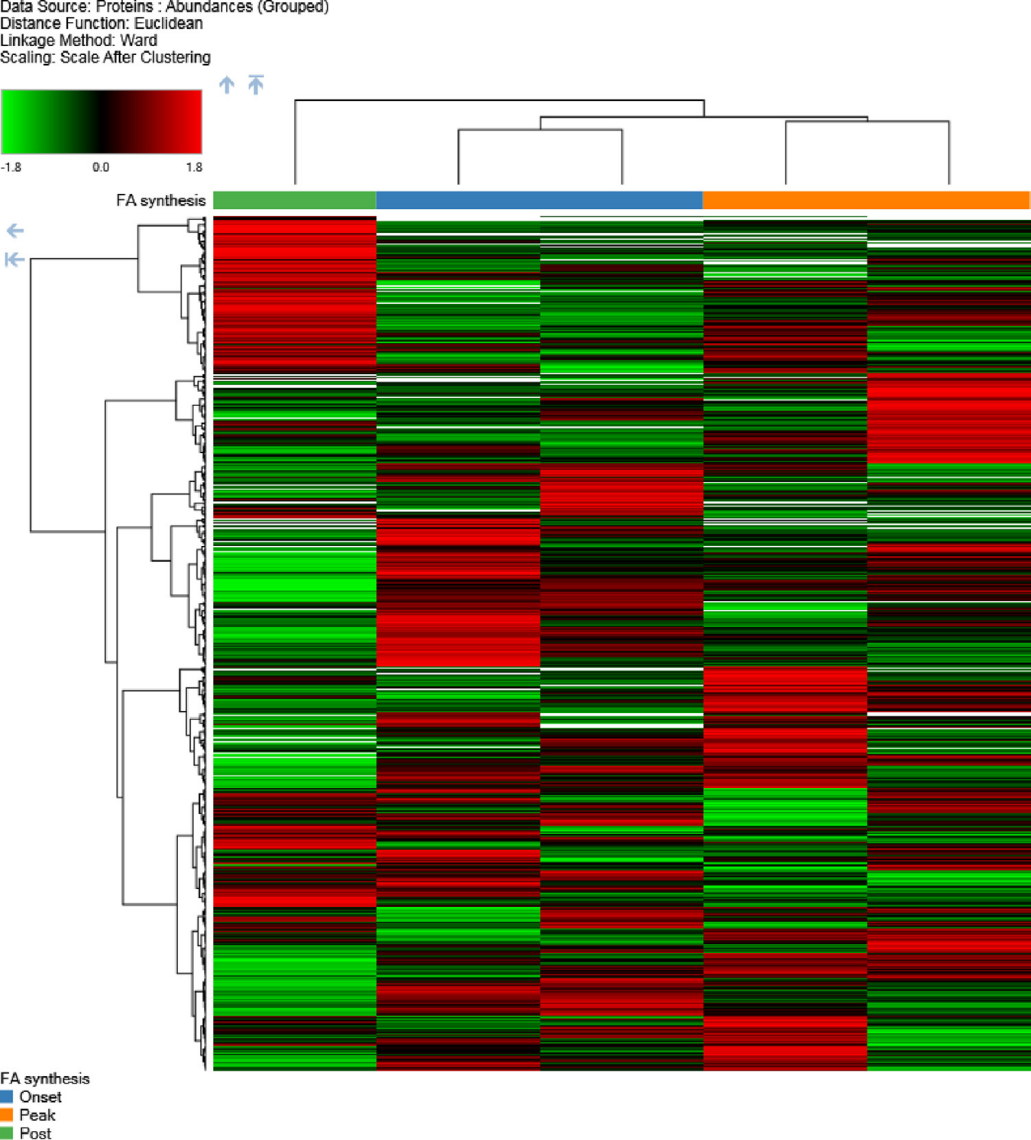
Heat map analysis of the identified proteins associated with lipid metabolism from *Elaeis guineensis* and *Elaeis oleifera* from five stages of fruit development.

Using the same datasets, a biological heat map of clusters from different development stages (onset, peak and post lipid biosynthesis) of *E. guineensis* and *E. oleifera* was generated (Fig. 4). The heat map depicted the datasets as clustered patterns which show an overview of the distribution of oil palm proteins represented according to their expressions.

**Fig. 5:**
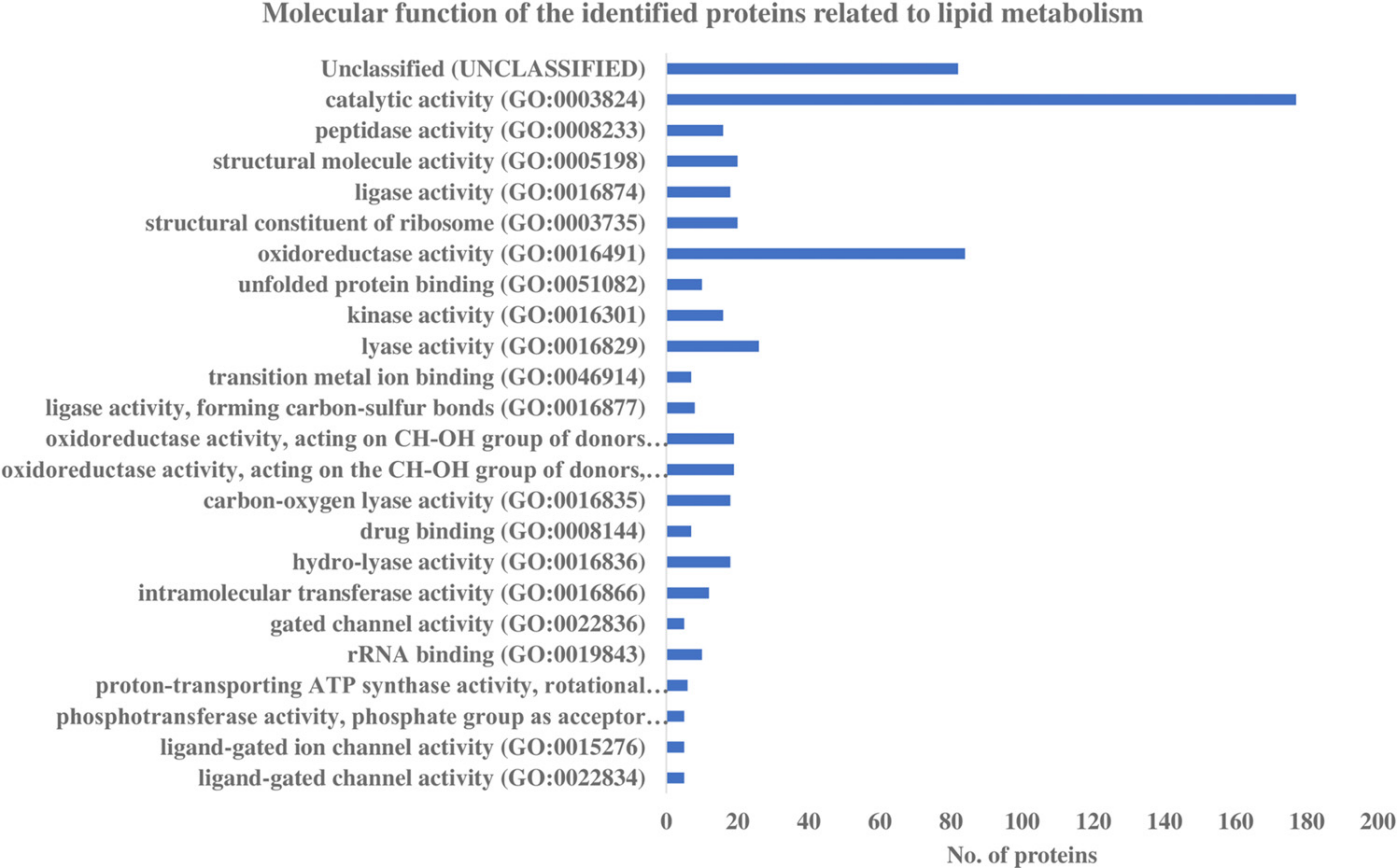
The molecular functions of the identified proteins associated with lipid metabolism from *Elaeis guineensis* and *Elaeis oleifera* based on the UniProt information available.

The identified proteins from *E. guineensis* and *E. oleifera* associated with lipid metabolism process (S2) were further classified according to their molecular functions based on the UniProt information available. Protein molecular functions shown in Fig. 5 revealed that 60.0% of the observed proteins were involved in catalytic activities while 28.5% were related to oxidoreductase activities or redox reaction. Pathway enrichment analysis demonstrated that the tricarboxylic acid cycle and 5-hydroxytryptamine degradation pathways were highly enriched (> 36-fold change at *p < 0.*05), based on the identified proteins (Table 1).

**Table 1:** Pathway enrichment analysis of the identified proteins associated with lipid metabolism from *Elaeis guineensis* and *Elaeis oleifera*.

PANTHER Pathways	Fold Enrichment	*p*-value
Tricarboxylic acid cycle (P00051)	36.04	1.57E-04
5-Hydroxytryptamine degradation (P04372)	36.04	1.57E-04
De novo pyrimidine ribonucleotides biosynthesis (P02740)	23.07	8.54E-03
Glycolysis (P00024)	17.3	2.24E-02
De novo pyrimidine deoxyribonucleotide biosynthesis (P02739)	16.48	2.64E-02
De novo purine biosynthesis (P02738)	9.2	4.76E-02
Heme biosynthesis (P02746)	11.28	3.16E-03
De novo purine biosynthesis (P02738)	9.2	3.16E-04
Unclassified (UNCLASSIFIED)	0.85	4.05E-19

## 2 Experimental Design, Materials, and Methods

### 2.1 Plant materials

Oil palm fruit bunches from *Elaeis guineensis* (commercial tenera), the MPOB-Nigerian tenera and *Elaeis oleifera* were sampled from Malaysian Palm Oil Board Research Stations located at Hulu Paka, Terengganu and Kluang, Johor, Malaysia. Mesocarps from random fruitlets were cut and frozen instantly in liquid nitrogen and kept at - 80°C until subsequent processes.

### 2.2 Protein extraction

Total proteins were obtained from the fruit mesocarps using method from Lau and co-workers [2]. About 10 g of sliced mesocarps of 1 mm in diameter (approximately) were ground in liquid nitrogen. 25 mL of cold acetone made of 10% trichloroacetic acid and 1 mM dithiothreitol, was added to the mesocarp powder. The suspension was centrifuged at 13,000 g for 10 min at 4°C (RA-300 rotor, Kubota 7820, Kubota Corporation, Tokyo, Japan). The washing step was repeated. Subsequently, 25 mL of cold 80% methanol comprising of 0.1 M ammonium acetate was used for washing and centrifuged as previously to obtain the pellet. Final washing involved 25 mL of cold 80% acetone. Mesocarp pellet obtained from centrifugation, as described previously, was re-suspended in 15 mL of buffer containing 1 M Tris-HCl, pH 8.3, 5 M NaCl, 0.7 M sucrose, 50 mM DTT, 1 mM EDTA and Roche protease inhibitors (one tablet). The mixture was homogenized with an ultrasonic homogenizer with amplitude of 40% for 30 s (total sonication time) (Fisherbrand Model 505, Pittsburgh, USA). Non-macerated materials were removed through filtering with two sheets of Miracloth (Calbiochem, EMB Millipore Corporation, Billerica, MA). 15 mL of 50 mM phenol saturated with Tris (pH 8.0) was then added to the resuspension and centrifuged at 15,000 g for 15 min at 4°C (RA-300 rotor, Kubota 7820). Proteins in the upper phase were extracted with the addition of 25 mL of cold ammonium acetate-saturated methanol. The mixture was incubated at -20°C for about 12 h. Protein pellet, obtained from centrifugation at 15,000 g for 15 min at 4°C (RA-300 rotor, Kubota 7820), was washed with 5 mL of cold methanol saturated with 0.1 M ammonium acetate methanol. Subsequent washes (three times) involved 5 mL of cold 80% acetone. The protein pellet was air-dried for about 5 min.

### 2.3 Digestion of proteins

Digestion was carried out in accordance to method from Lau and co-workers [3]. 50 µg of extracted proteins was re-suspended in 100 µL of 50 mM ammonium bicarbonate and 1 M urea. The proteins were reduced and alkylated using 100 mM tris(2-carboxyethyl)phosphine and 200 mM iodoacetamide, respectively. 1% (w/v) of sodium deoxycholate in 5 mM ammonium bicarbonate was used to improve the peptide solubility before the tryptic digestion using 1 µg of modified sequencing grade trypsin (Promega, Madison, WI, USA). The digestion was performed at 37°C for 16 h. After the peptide mixture was acidified with 0.5% formic acid, sodium deoxycholate precipitate was obtained through centrifugation at the speed of 14 000 g (RA-300, Kubota 7820) at room temperature for 15 min. Centrifugal evaporator (CentriVap Concentrator, Labconco, MO, USA) was used to remove the solvents and acids. The desiccated peptides were re-suspended in 100 µL of 0.1% formic acid. An Empore solid phase extraction disk (3M Purification, Inc., MN, USA), conditioned with acetonitrile and methanol, was inserted into the resuspension for incubation at room temperature for 4 h. Elution of the peptides from the disk was done using 50% ACN in 0.1% FA for 2.5 h in a sequential manner.

### 2.4 Liquid chromatography-mass spectrometry analysis

Peptides were reconstituted in 30 µL of 0.1% FA and 5% ACN. 2 µL of digests was loaded onto an Acclaim PepMap 100 C18 column (3 µm, 0.075 × 150 mm) (Thermo Scientific, MA, USA). The reverse phase column was equilibrated with 95% of 0.1% FA (mobile phase A) and 5% of ACN containing 0.1% FA (mobile phase B). Gradient of 5-35% mobile phase B in a total of 70 min, at a flow rate of 300 nL min^−1^, was applied for peptide elution. Separation of the peptides was done using an EASY-nano liquid chromatography (EASY-nLC) 1200 System (Thermo Scientific, MA, USA). An online Q Exactive Plus Hybrid Quadrupole-Orbitrap mass spectrometer system (Thermo Scientific, MA, USA) was used to generate the peptide ions with a spray voltage of 1800 V in positive mode. Precursor ion scan was conducted with a resolution of 70,000 and a mass range of *m/z* 310-1800. Precursors containing charge state from 2+ to 8+ were fragmented further. The fragmentation was done via collision-induced and high-energy collision-induced (CID and HCD) at a normalized energy of 28%, correspondingly. The resolution, isolation window and ion injection time was set at 17,500, 0.7 Da and 60 ms, respectively. Scanned precursor mass range was set at *m/z* 110-1800.

### 2.5 Data mining

Mass spectra of the peptides were acquired using Xcalibur (Ver. 4.1.31.9) (Thermo Scientific, MA, USA) and deconvoluted with Proteome Discoverer (Ver. 2.2) (Thermo Scientific, MA, USA) to create the peptide mass list. SEQUEST HT search engine, incorporated in the Proteome Discoverer, was used to match the generated mass list against two taxonomies in NCBI; *Elaeis guineensis* (Taxonomy ID is 51953, 35,972 sequences) and *Phoenix dactylifera* (Taxonomy ID is 42345, 33,101 sequences). Mass tolerance for the peptides and its fragments was fixed at 20 ppm and 0.5 Da, respectively. Trypsin was indicated as the digestion enzyme used, with up to two miscleavages were allowed during the search. Carbamidomethylation modification on cysteine residues was set as a static modification while variable amino acid modifications included deamidation (asparagine and glutamine residues) and oxidation (methionine residues). The mass list was also searched against a decoy database generated from the randomised protein sequences of the two taxonomies mentioned previously. The identified proteins must have at least a Rank 1 peptide and a false discovery rate of 1%, in order to be accepted. Spectra that matched to the sequences were further validated with Percolator algorithm (Ver. 2.04) using *q*-value at 1% false discovery rate. 12^th^ and 14^th^ week after anthesis were categorised as the onset of fatty acid biosynthesis; 16^th^ and 18^th^ week after anthesis as the peak of fatty acid biosynthesis; and 20^th^ week after anthesis as the post fatty acid biosynthesis. The groups were clustered using principal component analysis and heat map generation was done with the statistical analysis component in Proteome Discoverer. The clustering method used was a simple agglomerative hierarchical clustering method (UPGMA). Distance measure applied was Euclidean in logarithmic scale for rows. Distance between clusters were computed with Ward's method. Venn diagram was generated using the web-based software available at www.interactivenn.net. Enrichment pathway analysis was performed using PANTHER Overrepresentation Test (Released 20190711) (www.pantherdb.org). Fisher's Exact test was applied and the expression data analysis statistics used a Bonferroni correction for multiple testing.

## Appendix Supplementary materials

S1. List of identified proteins from all oil palm species (XLSX). Image, application 1

S2. List of proteins involved in lipid metabolism and differentially expressed (XLSX). Image, application 2

Image, application 3
